# Endogenous endophthalmitis due to *Escherichia coli*: a case report

**DOI:** 10.5935/0004-2749.2023-0066

**Published:** 2024-03-27

**Authors:** João Pedro Romero Braga, Victor C. F. Bellanda, Moises Moura de Lucena, Francyne Veiga Reis, Rodrigo Jorge

**Affiliations:** 1 Division of Ophthalmology, Faculdade de Medicina de Ribeirão Preto, Universidade de São Paulo, Ribeirão Preto, SP, Brazil

**Keywords:** Endophthalmitis, Escherichia coli, Escherichia coli infections, Eye infections, Bacterial, Sepsis, Vitrectomy, Anti-bac-terial agents/therapeutic use, Humans, Case reports

## Abstract

Endophthalmitis is a severe form of purulent inflammation caused by the infection of the intraocular tissues or fluids. This infection infrequently occurs through endogenous routes, which are often correlated with major risk factors. *Escherichia coli*, a gram-negative rod, can cause endophthalmitis through hematogenous spread. We here report a 59-year-old man who presented to our service with acute visual impairment in his left eye, preceded by floaters. He was taking sirolimus and azathioprine for a transplanted kidney, had undergone catheterization for bladder atresia, and had a history of recurrent *E. coli* urinary tract infections. On evaluation, the left eye exhibited visual acuity of hand motion, anterior chamber reaction (3+/4+), and intense vitritis (4+/4+) with white flake clusters, which prevented appropriate retinal evaluation. Pars plana vitrectomy was performed, and the culture yielded *E. coli*. The present case highlights the importance of identifying the signs and symptoms of infection early so that diagnosis and treatment of endophthalmitis can be promptly initiated.

## INTRODUCTION

Endophthalmitis is a severe inflammation caused by intraocular cavity infection. It is diagnosed on the basis of inflammatory signs in the anterior segment, which may be initially mild and limited to the anterior segment but become severe and affect the posterior segment. A lack of timely and adequate treatment of endophthalmitis can lead to irreversible visual loss^(1)^. Therefore, attention to early clinical signs is crucial.

Endophthalmitis is classified according to the infection route. Exogenous endophthalmitis occurs when the infectious agent is directly inoculated into the ocular cavity through intraocular surgery, penetrating trauma, or contiguous spread from adjacent tissues. Meanwhile, endogenous endophthalmitis occurs due to hematogenous spread from a remote infectious site^(1^,^2)^. Endogenous endophthalmitis is less common and is typically correlated with major risk factors, including recurrent urinary tract infections (UTIs), abdominal surgery, recent hospitalization, an indwelling catheter, and intravenous drug use. It occurs more frequently in patients of very young or advanced age, as well as those with diabetes mellitus, malignancies, or using immunosuppressive drugs^(1^,^2)^.

Gram-positive bacteria such as *Staphylococcus* and *Streptococcus* spp. are responsible for most global cases of endophthalmitis. Endophthalmitis caused by gram--negative bacteria, such as *Klebsiella* spp., occur more frequently in Asia^(1)^ and are uncommon in Brazil. Endophthalmitis caused by *E. coli* occurs most rarely, with only a limited number of cases being reported^(2^-^10)^. We here describe a case of endophthalmitis occurring due to *E. coli* septicemia and highlight the significance of detecting early signs of infection for prompt diagnosis and treatment.

## CASE REPORT

A 59-year-old male physician presented with acute visual impairment in his left eye, preceded by floaters. He had been using sirolimus and azathioprine for a transplanted kidney, had undergone catheterization for bladder atresia, and had experienced recurrent *E. coli* UTI. He had been hospitalized and received broad-spectrum antibiotics, in addition to voriconazole, during his last UTI. One week after admission, he had tested positive for COVID-19, evolving with severe pulmonary impairment, and had remained hospitalized for 47 days. Within a few days after discharge, he developed another episode of UTI, caused by *Proteus mirabilis*. The urinary symptoms recurred after treatment, and two weeks later, he developed floaters, photophobia, and poor visual acuity (VA). An ophthalmologist prescribed him topical corticosteroids and mydriatic drops and referred him to our department.

**Figure 1:**
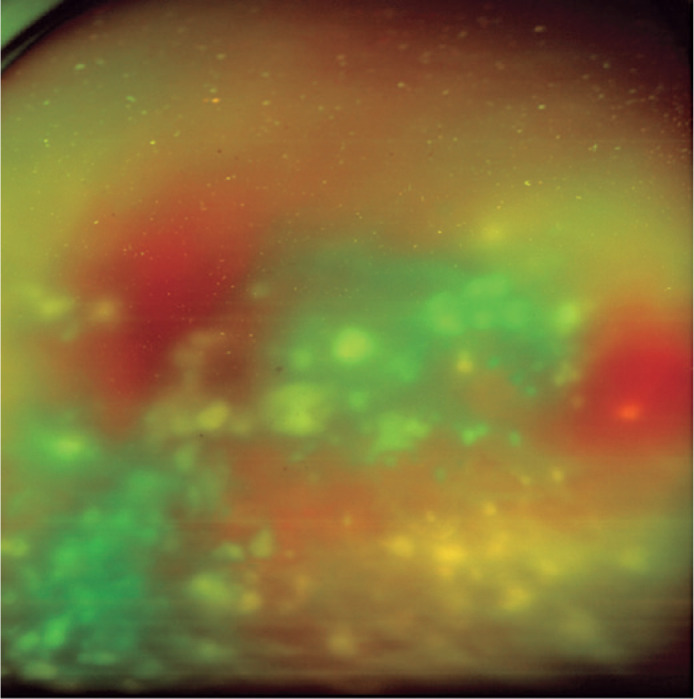
Baseline fundus picture of the left eye displaying intense vitritis and diffuse white lesions in the vitreous cavity.

Four months after hospitalization, fever and urinary symptoms recurred. Urine culture was positive for *E. coli*, and his general practitioner initiated systemic ciprofloxacin. The patient was then examined at our clinic with a VA of 20/25 in the right eye (OD) and hand motion in the left eye (OS). Biomicroscopy revealed trace nuclear cataracts and the absence of anterior chamber reaction in the OD, while cortico-nuclear cataracts (2+/4+) and anterior chamber reaction (3+/4+) without conjunctival hyperemia were observed in the OS. Fundoscopy was unremarkable in the OD but exhibited intense vitritis (4+/4+) with some white flake clusters in the OS, which thus prevented appropriate retinal evaluation (Figure 1).

Pars plana vitrectomy with a 23-gauge needle was promptly performed for the OS. A vitreous culture was obtained at the start of surgery, followed by intravitreal injection of ceftazidime, vancomycin, and amphotericin at the end of the procedure. After the surgery was completed, moxifloxacin/dexamethasone (Vigadexa^®^) and mydriacyl eye drops as well as oral fluconazole and amoxicillin-clavulanate were prescribed, as recommended by the attending physician. A few days later, the culture was again positive for *E. coli*.

**Figure 2:**
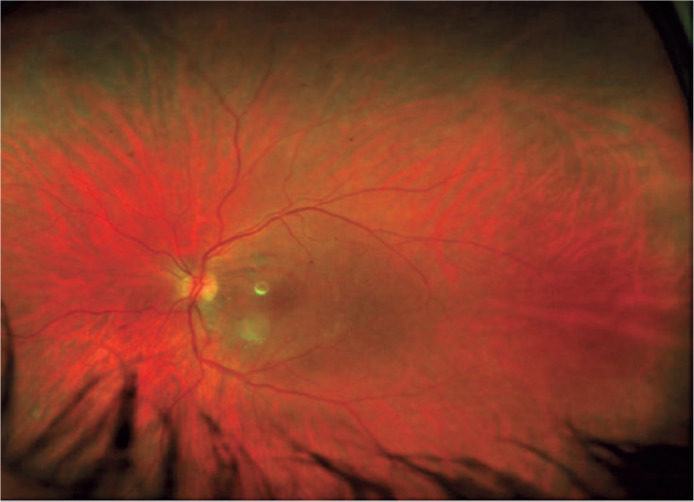
Fundus picture of the left eye after pars plana vitrectomy and phacoemulsification, with final visual acuity of 20/40.

The patient showed no infection recurrence after vitrectomy, and the retina remained attached, although the VA in the OS was 20/200 because of posterior subcapsular cataracts. Phacoemulsification improved the VA to 20/50, but the patient developed cystoid macular edema, which decreased the VA to 20/80. However, with the use of a retarded release dexamethasone implant, the final VA was stabilized to 20/40 in the affected eye (Figure 2).

## DISCUSSION

Although endogenous endophthalmitis commonly occurs in immunocompromised patients, it has also been reported in healthy adults^(5)^. It can occur at any age and has no sex predilection. Our patient was immunosuppressed, had received permanent catheterization because of bladder atresia, and had a history of kidney transplantation and recurrent UTI, which likely contributed to the rare etiology of this case.

Decreased vision, eye pain, discomfort, and hyperemia, are common, but not universal, symptoms of endophthalmitis. Systemic symptoms such as fever are absent in exogenous cases but are often noted in endogenous cases^(6^,^7)^. Our patient had poor VA, fever, and urinary symptoms, which could be misinterpreted as UTI alone.

Vitrectomy, intravitreal antibiotics, and systemic therapy for the underlying infection are often included as standard treatment for endogenous endophthalmitis^(1^,^2)^. The prognosis of this condition is poor in most patients; in a series of 75 patients (89 eyes), only 41% of the eyes recovered 20/200 vision or better, whereas 19% were enucleated or eviscerated^(5)^. Vitrectomy has been found to be associated with a better visual prognosis and lower rates of evisceration or enucleation^(5)^.

To date, only a few cases of *E. coli* endogenous endophthalmitis have been reported^(3^-^10)^. The involved patients generally had diabetes and had the urinary tract as the most frequent primary infection site, as in the present case. Other sites affected were the conjunctiva and gallbladder^(5^,^6)^. *E. coli endophthalmitis* has a rapid and devastating course; of the 4 bilateral and 14 unilateral reported cases, only 7 eyes could be saved despite intensive therapy. One eye achieved a VA of 20/50, whereas all other recovered eyes had no better VA than hand motions or light perception^(3^-^10)^. The uncommon, good outcome could not have been attained in the absence of prompt identification and treatment according to the vitreous culture.

Endogenous endophthalmitis may occur through hematogenous spread from remote sites, particularly when the patient has transient bacteremia or long--term catheter-related fungemia. The primary source of infection in our patient was the urinary tract, as noted in other reports. Furthermore, the COVID-19 infection acquired during hospitalization may have helped the hematogenous spread to the eye by damaging the vascular endothelium.

Early identification of infectious signs and a thorough clinical assessment are fundamental for prompt diagnosis and treatment, thereby leading to a better visual prognosis. Despite being rare, *E. coli* should be considered a potential infectious agent in immunocompromised patients, especially those with UTIs.
